# Early Detection of Lewis Lung Carcinoma Tumor Control by Irradiation Using Diffusion-Weighted and Dynamic Contrast-Enhanced MRI

**DOI:** 10.1371/journal.pone.0062762

**Published:** 2013-05-02

**Authors:** Jason Chia-Hsien Cheng, Ang Yuan, Jyh-Horng Chen, Yi-Chien Lu, Kuan-Hung Cho, Jian-Kuen Wu, Chien-Jang Wu, Yeun-Chung Chang, Pan-Chyr Yang

**Affiliations:** 1 Graduate Institute of Oncology, National Taiwan University College of Medicine, Taipei, Taiwan; 2 Graduate Institute of Clinical Medicine, National Taiwan University College of Medicine, Taipei, Taiwan; 3 Department of Radiology, National Taiwan University College of Medicine, Taipei, Taiwan; 4 Graduate Institute of Biomedical Electronics and Bioinformatics, National Taiwan University College of Electrical Engineering and Computer Science, Taipei, Taiwan; 5 Department of Oncology, National Taiwan University Hospital, Taipei, Taiwan; 6 Department of Medical Imaging, National Taiwan University Hospital, Taipei, Taiwan; 7 Department of Internal Medicine, National Taiwan University Hospital, Taipei, Taiwan; 8 Instrumentation Resource Center, National Yang-Ming University, Taipei, Taiwan; 9 Institute of Electro-Optical Science and Technology, National Taiwan Normal University, Taipei, Taiwan; National Yang-Ming University, Taiwan

## Abstract

**Purpose:**

To investigate the correlation between diffusion-weighted (DW) and dynamic contrast-enhanced (DCE) magnetic resonance imaging (MRI) derived parameters and radioresponsiveness of Lewis lung carcinoma (LLC) tumor.

**Materials and Methods:**

LLC tumor growth in C57BL/6 mouse limb was used for the experiment. The tumors were irradiated with 10 Gy×5, or 30 Gy×2 vs. sham irradiation. Fourteen tumors were subjected to DW-MRI and DCE-MRI pre-radiotherapy and weekly imaging after radiotherapy. The temporal changes in apparent diffusion coefficient (ADC) and DCE-MRI derived parameters (K^trans^, k_ep_, v_e_, and v_p_) were correlated with tumor size, and were histologically compared with CD31 staining of resected tumors.

**Results:**

The 10 Gy×5 dose inhibited tumor growth for a week, while 30 Gy×2 controlled tumor growth for a 3-week observation period. One week after radiotherapy (week 2), irradiated tumors showed significantly higher values of ADC than untreated ones (10 Gy×5, p = 0.004; 30 Gy×2, p = 0.01). Significantly higher values of v_e_ were shown earlier by 30 Gy×2 vs. sham (p = 0.01) and 10 Gy×5 vs. sham irradiation (p = 0.05). Sustained higher v_e_ from 10 Gy×5 compared to sham irradiated tumors was evident at week 3 (p = 0.016) and week 4 (p = 0.046). A 13.8% early increase in ADC for 30 Gy×2 tumor group (p = 0.002) and a 16.5% increase for 10 Gy×5 group were noted (p = 0.01) vs. sham irradiation (which showed a 2.2% decrease). No differences were found for K^trans^, k_ep_, or v_p_. Both radiotherapy groups demonstrated significant reduction in microvessel counts.

**Conclusion:**

Early increase in ADC and v_e_ correlated with tumor control by irradiation.

## Introduction

Radiotherapy (RT) has been an essential treatment modality for approximately half of all cancer patients [1]. Adequate radiation dose is one of the important prognosticators for disease control in a variety of malignancies [2]. Dose escalation has proven effective in treatment outcome from randomized clinical trials [3]. Suboptimal dose is associated with unsatisfactory tumor control and unwanted effects, such as metastasis [4].

A mouse model has been established in which primary Lewis lung carcinoma (LLC), implanted in thighs eradicated by irradiation (five 10-Gy fractions), was followed by the development of pulmonary metastasis [5]. Using this model, our team demonstrated that metastasis were likely derived from sub-lethal irradiation of the primary tumor and its activated signaling cascade [6].

Imaging tools, such as computed tomography (CT), magnetic resonance imaging (MRI), and positron emission tomography (PET), are non-invasive methods that provide staging information and monitor the treatment response of malignant disease. The original response evaluation criteria in solid tumors (RECIST) defined the response to treatment in terms of alteration of tumor size only [7]. The RECIST criteria have recently been revised with the inclusion of PET as one of the biomarkers [8].

Obstacles, however, remain regarding confirmation of lethality or viability of the treated tumors after chemotherapy, target therapy, and radiotherapy. It takes weeks, or sometimes months, for follow-up examinations to identify change in disease progression, primarily by morphological criteria [9]. Such latency prohibits the timely salvage treatment of viable diseased tissue.

Diffusion-weighted MRI (DW-MRI), which generates an apparent diffusion coefficient (ADC), has been shown to correlate with cell density measures in cancer models [10], [11]. Increased ADC has been reported to be an indicator of early tumor response to chemotherapy or target therapy [12]. Dynamic contrast-enhanced MRI (DCE-MRI) provides the parameters of K^trans^ (volume transfer constant), k_ep_ (rate constant of backflux from extravascular extracellular space [EES] to plasma), v_e_ (total volume of EES per unit of tissue), and v_p_ (total blood plasma volume). These parameters display sensitive pathophysiological characteristics and changes in tumor vasculature after injection of contrast agents used for kinetic distribution within the region of interest (ROI) [13].

We, therefore, investigated the time-dependent dynamics of both DW-MRI and DCE-MRI using our established LLC mouse model with two different doses of radiation. We hypothesized DW-MRI and DCE-MRI parameters would correlate with early response of LLC tumor treated by irradiation.

## Materials and Methods

### Cell Culture

LLC cells were grown at 37°C in a humidified atmosphere of 5% CO_2_/95% air in DMEM containing 10% heat-inactivated fetal bovine serum plus penicillin-streptomycin under sterile tissue culture conditions.

### Mouse Model and Tumor Irradiation

This study was carried out in strict accordance with the recommendations in the Guide for the Care and Use of Laboratory Animals of the National Institutes of Health. The protocol was approved by the Committee on the Ethics of Animal Experiments of the National Taiwan University College of Medicine (Permit Number: 20120092). All surgery was performed under sodium pentobarbital anesthesia, and all efforts were made to minimize suffering.

Male C57BL/6 mice, 5- to 6-weeks-old, (National Taiwan University Animal Center, Taipei, Taiwan) were used in our study. For each experiment, 1×10^6^ cells from one of several different LLC cell lines were injected subcutaneously into the right hind limb of each mouse. At 8 days after implantation, mice were immobilized in a customized harness that left the right hind leg exposed. The remainder of the body was shielded by 5 times the half-value-thickness of lead. A linear accelerator (Siemens Mevatron, Siemens, Concord, CA) with 6-MV photon was used to irradiate the primary tumor with 50 Gy (five 10-Gy daily fractions, at the dose rate of 1 Gy/minute on days 8–12), or 60 Gy (two 30-Gy fractions on day 8 and day 10). The study design was based on our previous work [6] on this tumor model with different morphological tumor control by these two radiation doses at weekly time points. On day 28, we sacrificed the mice after intraperitoneal or intravenous injection of potassium chloride, dissected the thigh tumors, and prepared them for the histological evaluation. In total, five, 6, and 6 mice from the groups of sham irradiation, 10 Gy×5, and 30 Gy×2, were imaged at four time points, including pre-RT (week 1), 1 (week 2), 2 (week 3), and 3 (week 4) weeks after RT, respectively.

### MRI Techniques

The 7T animal scanner (BioSpec 70/30 USR, Bruker AXS, Inc, Madison, WI), equipped with phase-array rat brain coil (Bruker), was used to acquire DCE-MRI for all experiments. MR parameters included repetition time (TR) of 100.1 ms, echo time (TE) of 3.8 ms, flip angle of 40 degrees, number of excitations (NEX) = 9 slices, field of view (FOV) = 35 mm^2^, slice thickness = 1 mm, intersection gap = 0 mm, matrix size = 256×192, in-plane resolution = 137×183 µm, and scanning time of 14.4 seconds per acquisition. There were a total of 60 acquisitions. Contrast medium was injected through the orbital cavity at the end of fifth acquisition (i.e., 72 seconds after the beginning of first acquisition).

DCE-MRI images were analyzed using the Tofts model [14], [15] and commercial software (Apollo Medical Imaging Technology Pty Ltd, Melbourne, Australia). After the cine perfusion images from DCE-MRI were registered, contours were manually drawn around each tumor. DCE MR parameters included K^trans^, k_ep_, v_e_, and v_p_. Tumor volume was obtained by summation of data from all slices containing the tumor. The analysis of DCE-MRI was done by commercial software and the analysis of DW-MRI was done by home-made program using MATLAB. The calculation of ADC is based on
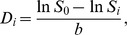
Where i = read, phase, or slice direction, and D_i_ is the apparent diffusion coefficient (ADC) for each direction. S_0_ is the signal from b0 image, S_i_ is the DW signal, and b is the diffusion sensitivity. After the ADC values for each of the three directions were calculated, the mean diffusivity (MD) could be obtained by



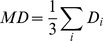



DW images were acquired using a pulsed-gradient spin-echo segmented echo planar imaging sequence with the following parameters: TR = 3000 ms, TE = 29.3 ms, in-plane resolution = 273×273 µm, slice thickness = 1–1.5 mm, intersection gap = 0 mm, number of segment = 4, NEX = 5, and b value of 700 s/mm^2^. ADC maps were generated from DW images using the b value. For DW-MRI, 9 slices were acquired the same as DCE-MRI for the comparison between DW-MRI and DCE-MRI. Three diffusion directions (read, phase, and slice directions) were used for DW-MRI.

### Histological Evaluation

After fixation, tumor tissues were embedded in paraffin blocks. Sections (5 µm) were cut and stained with hematoxylin and eosin (H&E) for histopathological evaluation, and were embedded in ornithine carbamyl transferase (OCT) and frozen to −80°C. Angiogenesis phenotypes of tumor-associated microvessels in ectopic tumor xenografts were evaluated using rat anti-mouse CD31 antibody.Sections were stained with rat anti-mouse monoclonal antibody CD31 (1∶50 dilution, clone MEC13.3, PharMingen, BD Pharmingen, San Diego, CA) (BD Biosciences, Franklin Lakes, NJ) for mouse endothelial cell staining. After incubation with primary antibody overnight, slides were washed and incubated with rabbit anti-rat IgG antibody (Millipore,Billerica, MA) for 60 minutes and then with Streptaviden-peroxidase (Invitrogen Ltd., Paisley, UK) for 60 minutes. The color was developed by incubating the slides with Diaminobenzidine (DAB) substrate kit (Zymed Laboratories Inc, San Francisco, CA) for 20 minutes. Counterstaining was prepared using Hematoxylin, giving a blue background. The capillaries surrounding alveoli of normal mouse lung tissue were used as positive controls for anti-CD31 staining. Negative controls were the sections stained without the use of primary antibodies.

The distribution and morphology of microvessels in each tumor were evaluated under microscopy. Brown immunostained endothelial cell clusters, that were clearly separated from one another, were considered a single microvessel. Microvessels in the area of most intense neovascularization were counted in three randomly chosen 200X magnification fields. The average of the three readings was defined as the microvessel count (MVC).

### Statistical Analysis

An ROI was manually drawn on each slice of tumor at each time point by a single experienced radiologist (YCC). The average signal intensity was obtained from the parametric values of the ROIs from each mouse at each time point. The same ROI was applied to both the DCE-MRI and DW-MRI data. All imaging data acquired at all the time points were included for the analysis.

Student-*t* test was used to determine significant differences between each treatment group (both absolute and percent change data). A *p* value less than 0.05 was considered statistically significant.

## Results

**Figure 1:**
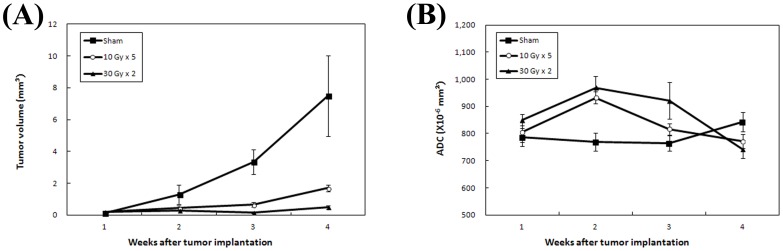
Plots of tumor volume and apparent diffusion coefficient (ADC) for each of the treatment groups. (A) Tumor volumes and (B) ADC of mice in three treatment groups were shown at each time point from week 1 (baseline) to week 4. Data presented were the mean ± SEM.

The tumors in the three treatment groups showed different growth velocities from the pre-RT baseline to week 4, with the largest growth in sham irradiated group, transient tumor growth inhibition with 10 Gy×5, and sustained control of tumor growth with 30 Gy×2 (Fig. 1). Enlarging areas of central tumor necrosis were associated with higher ADC values. Statistically, the increase in ADC values was significant when comparing 10 Gy×5 vs. sham groups (*p* = 0.004), and 30 Gy×2 vs. sham groups (*p* = 0.01) at week 2, but not for the other time points. Mean absolute ADC values for the treatment groups are listed in Table 1.

**Table 1:** Average absolute apparent diffusion coefficient (ADC) values (×10^−6^ mm^2^), K^trans^, k_ep_, v_e_, v_p_, and their standard deviations of mice in three treatment groups at each time point from week 1 (baseline) to week 4.

	Week 1	Week 2	Week 3	Week 4
ADC				
Sham	786.2±66.6	768.8±66.9	764.3±55.3	842.9±50.4
10 Gy×5	805.6±82.3	932.1±49.0	816.8±46.0	772.3±58.0
30 Gy×2	849.8±52.5	968.9±104.2	922.0±163.8	742.2±80.8
K^trans^				
Sham	118.7±117.0	28.6±12.3	46.0±38.1	9.5±7.1
10 Gy×5	106.6±106.5	48.6±33.3	46.4±16.7	17.7±5.8
30 Gy×2	34.7±18.5	62.8±35.7	31.8±22.4	19.4±11.5
k_ep_				
Sham	980.4±1017.6	364.5±215.1	509.0±396.4	365.6±126.4
10 Gy×5	1411.9±1442.2	429.0±354.6	355.7±248.5	276.9±63.8
30 Gy×2	321.9±265.5	430.3±579.0	526.4±530.1	261.3±136.4
v_e_				
Sham	192.5±141.3	91.8±36.3	75.2±34.1	28.4±23.7
10 Gy×5	155.0±98.1	177.1±79.1	183.5±75.0	82.3±24.6
30 Gy×2	183.3±106.8	290.9±131.2	125.0±69.6	80.0±38.5
v_p_				
Sham	88.1±41.5	20.4±7.5	23.1±13.1	10.6±11.8
10 Gy×5	116.2±28.1	135.2±54.3	72.7±41.7	22.3±11.7
30 Gy×2	133.9±51.8	116.4±104.8	130.7±106.1	29.5±30.7

**Figure 2:**
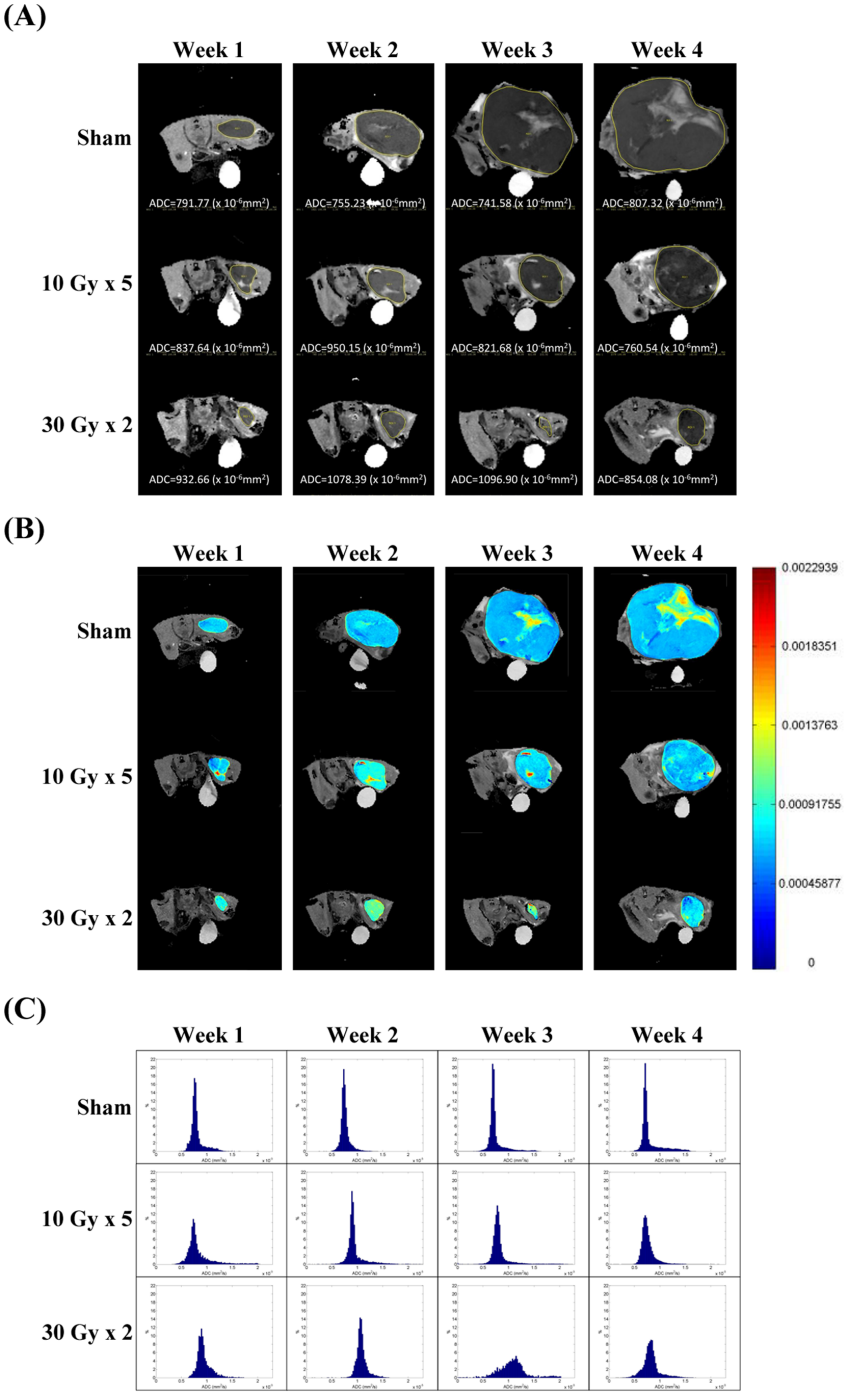
MR, apparent diffusion coefficient (ADC) parametric map, and normalized histogram from representative animals. (A) ADC mapping on diffuse-weighted images, (B) ADC parametric maps, and (C) normalized histograms of ADC values of one representative mouse from each treatment group at the indicated time points.

The DW images and parametric maps of one representative mouse from each treatment group were shown in Figs. 2A and 2B. Normalized histograms of the three mice indicated a different peak shift for the ADC changes between the treatment groups (Fig. 2C). The peak shifts toward higher ADC values were transiently shown at week 2 for the mouse treated with 10 Gy×5, and more evident at week 2 and week 3 for the mouse treated with 30 Gy×2.

**Figure 3:**
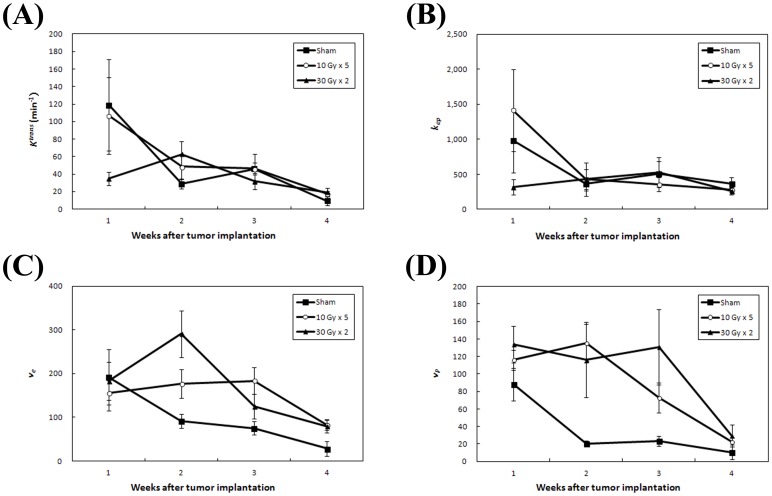
Plots of parametric changes of dynamic contrast-enhanced magnetic resonance imaging (DCE MRI) for each of the treatment groups. The changes of DCE MRI parameters, (A) K^trans^, (B) k_ep_, (C) v_e_, and (D) v_p_, of mice in three treatment groups were shown at each time point from week 1 (baseline) to week 4. Data presented were the mean ± SEM.

The group changes at each time point for the four DCE-MRI parameters, K^trans^, k_ep_, v_e_, and v_p_, are displayed in Fig. 3. K^trans^ and k_ep_ data from the different treatment groups are shown in a similar fashion. Neither K^trans^ and k_ep_ correlated with tumor growth.

Significantly higher v_e_ values were shown by 30 Gy×2 group compared to sham group (*p* = 0.01) and 10 Gy×5 compared to sham groups (*p* = 0.05) at week 2. In addition, the effect was sustained longer by the 10 Gy×5 group compared to sham groups at week 3 (*p* = 0.016) and week 4 (*p* = 0.046). However, a significant difference in v_p_ was seen only between 10 Gy×5 and sham groups at week 2 (*p* = 0.001) but not between 30 Gy×2 and sham groups. Mean absolute values of K^trans^, k_ep_, v_e_, and v_p_ for the treatment groups are listed in Table 1.

**Figure 4:**
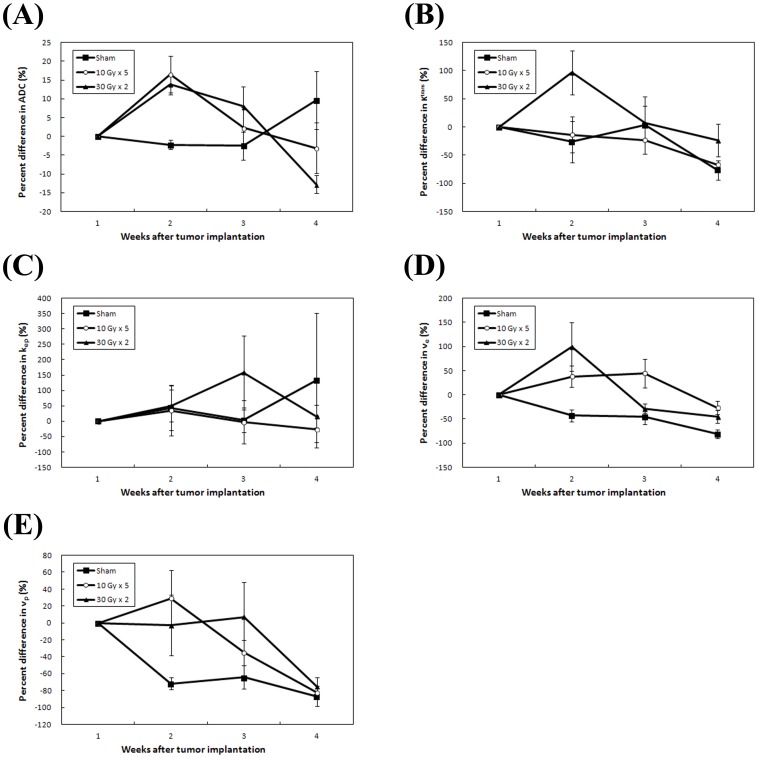
Plots of percent change in functional MRI parameters for each of the treatment groups. Percent changes of (A) apparent diffusion coefficient (ADC), (B) K^trans^, (C) k_ep_, (D) v_e_, and (E) v_p_, of mice in three treatment groups were shown at each time point from week 1 (baseline) to week 4. Data presented were the mean ± SEM.

The percent change between the treatment groups are displayed in Fig. 4. At week 2, mice treated with 30 Gy×2 showed a mean ADC increase of 13.8%, as compared to 16.5% in mice treated with 10 Gy×5, and −2.2% in sham irradiated mice. The differences were significant between 10 Gy×5 and sham groups (*p* = 0.01), and between 30 Gy×2 and sham groups (*p* = 0.002). At weeks 2, the mean differences in v_e_ change ratios were 99.1% in 30 Gy×2, 37.8% in 10 Gy×5, and −43.1% in sham groups, respectively. The differences were significant between 30 Gy×2 and sham group (p = 0.03) as well as 10 Gy×5 and sham group (*p* = 0.01). No significant differences were found between groups at these time points for either K^trans^, k_ep_, or v_p_.

**Figure 5:**
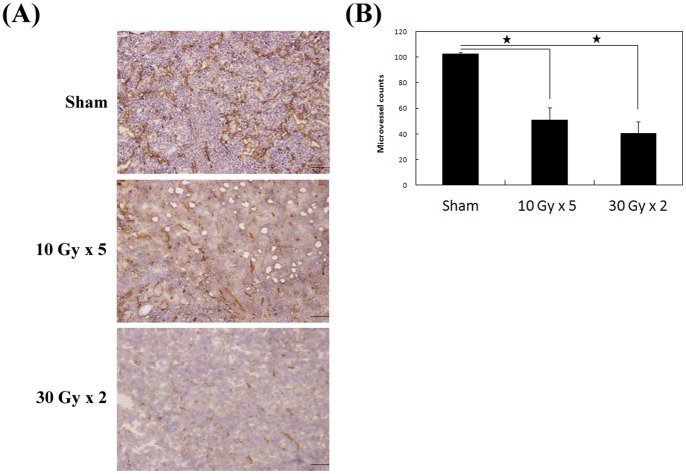
Histological staining of tumor tissue for quantifying the angiogenesis-related parameters. (A) Anti-CD31 staining of the sample sections from mice of three treatment groups (200X). (B) Microvessel counts in one high power field (200X) of mice in each treatment group. Columns, mean; Bars, S.D. *, *P*<0.01.

As shown in Fig. 5A, there were significant histologic differences in the MVCs among the three groups. The reduction in MVCs was detected by anti-CD31 staining, with significant differences found in sham versus 10 Gy×5 (*p* = 0.006) and sham versus 30 Gy×2 (*p* = 0.0003), but not in 10 Gy×5 versus 30 Gy×2 groups (*p* = 0.17) (Fig. 5B).

## Discussion

Unsatisfactory tumor control using RT potentiates locoregional disease progression and distant metastasis [16]. Higher dose intensity by stereotactic body RT, with fewer fractions and larger fractional dose, has also been used for certain radioresistant malignancies [17]. After RT, however, it takes weeks or months to evaluate the irradiated tumor for any morphological change or indirect evidence of lethality or viability [18]. Oncologists have become dissatisfied with such delays which limit timely rescue action and the non-functional criteria used to determine a therapeutic effect. The need for more sensitive imaging tools, such as DCE-MRI and DW-MRI, is required for earlier evaluation of tumor response to RT [19]. However, it remains unclear whether the survival would be truly improved with the additional therapeutic options by showing early imaging signals or even identifying viable component after RT.

With the established LLC mouse model [6], we were able to give two RT doses (10 Gy×5 and 30 Gy×2) for both short and long periods of tumor control. The dose of 10 Gy×5 inhibited the tumor growth within 1–2 weeks after RT, whereas 30 Gy×2 suppressed the tumor for 3 weeks. Notably, the tumor size difference between treatment groups was not significant immediately after RT (week 2), which would comprise the early timing of salvage treatment. Besides, there has not been any necrosis or other features currently used to predict later recurrence at this early timing after RT. We demonstrated a significant increase in ADC and v_e_ values at week 2 between RT dose groups, indicating a therapeutic effect was identified early using DW-MRI and DCE-MRI.

DW-MRI has been developed to quantify the Brownian motion of water molecules [10]. Higher cell density and intact tissue structure are characterized by a lower ADC value due to the decreased random motion of water. Tissue loss with increased water mobility generates higher ADC values. Higher ADC values are expected to correspond to cell death with loss of cell membrane integrity and reduction in tumor cell density after effective treatment [12].

We demonstrated an early increase in both absolute ADC values and percent change for irradiated compared untreated tumors, before the change in tumor size became apparent. Similar findings were reported by Yabuuchi et al. [20], who demonstrated a correlation between early ADC increase and final tumor size reduction as well as improved survival in lung cancer patients. Early percent change in ADC and related tumor responsiveness were also shown by Sun et al. in patients with various malignancies [21]. Significant increases in ADC values corresponding to diminished vasculature and cell death were found at three days in lung cancer xenografts treated with targeted drugs by Loveless et al. [22]. University of Michigan group similar demonstrated the early increase in normalized ADC prior to tumor volume change in their brain tumor model treated by RT, gemcitabine, and temozolomide [23]. All these studies (including ours) indicate the usefulness of early ADC increase as a biomarker for tumor response to effective therapy.

DCE-MRI has been widely used to assess vascular perfusion and permeability by pharmacokinetic modeling of tissue in patients with different cancers [19]. Among the parameters derived from DCE-MRI in our study, v_e_ was the only parameter significantly higher in the RT groups compared to the sham group. Similar findings were reported by Chikui et al. who showed that oral cancer patients treated by chemoradiotherapy had higher v_e_ in the responders [24]. Kim et al. showed that an early increase in v_e_ (but not its pretreatment level) predicted response to chemoradiotherapy for cervical cancer patients [25].

We hypothesized that increased v_e_ composition is the result of expanded EES of the irradiated tumor. This biological interaction is similar to the increased water diffusion reflected by higher ADC values in RT treated mice. These reactions signal LLC cell injury and death by RT. The final outcome was shown by decreased vascularity within the microenvironment of irradiated tumor as reflected by decreased MVCs. However, divergent conclusions, primarily regarding baseline and change in K^trans^, were proposed by other studies using different treatment modalities and cancer types [26], [27], [28]. The dominant MRI findings might vary between animal xenografts and patients, and between different malignancies [13].

Our study had several limitations. To determine a sufficient size difference between two different-dose groups, an observational period of 4 weeks was required. For fast growing tumors, this time frame did not allow an adequate number of surviving mice in the sham irradiated group for serial imaging studies. The small number of mice may have introduced uncertainty into the data. Besides, the differences in most of the functional MRI parameters for the irradiated tumor were not significant between the two RT dose groups. Despite the differently activated metastatic cascades caused by these two dose schedules (as previously reported by our team), the DW-MRI or DCE-MRI were not sensitive enough to demonstrate a corresponding difference in signals from the irradiated tumors. The high dose (30 Gy×2) used in this study did not lethally control the tumor at the end of the 4-week observation. It partly explained the late decrease in ADC. Moreover, by using a controlled animal model, the starting tumor size and tumor location within the mice were similar. The analyses were drawn more from temporal changes rather than baseline characteristics. The better model with the long-term control of LLC tumor by irradiation might further disclose the true value of MRI biomarkers. Notably, the early changes of MRI parameters at week 2 were not coincident to the histological microvessel staining at the end of week 4 for the longitudinal data collection from the same mouse. In this study, the scanning time as short as possible for the weak mice bearing tumors did not allow the measurements of more indices by DW-MRI, which could be the important focuses of future work.

### Conclusion

An early increase in ADC values on DW-MRI and in v_e_ on DCE-MRI (either by absolute value or percent change) correlated with final LLC tumor control and histological reduction in MVC by RT.
